# Insights into Resistance to Fe Deficiency Stress from a Comparative Study of *In Vitro*-Selected Novel Fe-Efficient and Fe-Inefficient Potato Plants

**DOI:** 10.3389/fpls.2017.01581

**Published:** 2017-09-13

**Authors:** Georgina A. Boamponsem, David W. M. Leung, Carolyn Lister

**Affiliations:** ^1^School of Biological Sciences, University of Canterbury Christchurch, New Zealand; ^2^The New Zealand Institute for Plant and Food Research Limited, Canterbury Agriculture and Science Centre Lincoln, New Zealand

**Keywords:** chlorosis, Fe-deficiency, ferritin, iron-regulated transporter, stress resistance

## Abstract

Iron (Fe) deficiency induces chlorosis (IDC) in plants and can result in reduced plant productivity. Therefore, development of Fe-efficient plants is of great interest. To gain a better understanding of the physiology of Fe-efficient plants, putative novel plant variants were regenerated from potato (*Solanum tubersosum* L. var. ‘Iwa’) callus cultures selected under Fe deficient or low Fe supply (0–5 μM Fe). Based on visual chlorosis rating (VCR), 23% of callus-derived regenerants were classified as Fe-efficient (EF) and 77% as Fe-inefficient (IFN) plant lines when they were grown under Fe deficiency conditions. Stem height was found to be highly correlated with internodal distance, leaf and root lengths in the EF plant lines grown under Fe deficiency conditions. In addition, compared to the IFN plant lines and control parental biotype, the EF plants including the lines named A1, B2, and B9, exhibited enhanced formation of lateral roots and root hairs as well as increased expression of ferritin (*fer3*) in the leaf and iron-regulated transporter (*irt1*) in the root. These morphological adaptations and changes in expression the *fer3* and *irt1* genes of the selected EF potato lines suggest that they are associated with resistance to low Fe supply stress.

## Introduction

Iron (Fe) is the fourth most abundant element in the Earth's crust, but about 30% of the world's cultivated soils are classified as calcareous and deficient in Fe for plant growth (Guerinot and Yi, 1994; Barton and Abadía, 2006; Hansen et al., 2006; Kim and Guerinot, 2007). The principal cause of Fe deficiency is that Fe occurs mainly in the form of water-insoluble hydroxides and oxides and/or carbonates–bicarbonates complexes that are not bioavailable for uptake by plant roots (Guerinot and Yi, 1994; Hell and Stephan, 2003; Zuo and Zhang, 2011). Other factors have also been linked to Fe deficiency. These include soil salinity, low temperature, high pH, carbonates, high moisture content, low water drainage, soil bulk density, high nitrate concentration, interactions of Fe with other soil minerals, landscape position across and within the same farmland (Hansen et al., 2006; Zuo and Zhang, 2011; Vasconcelos and Grusak, 2014). Reduced Fe^2+^availability results in chlorosis, stunted growth, reduction in crop quality and yields and a decrease in nutritional value of many crops (Naik et al., 1990; Abadia et al., 2011; Hindt and Guerinot, 2012; Bert et al., 2013; García-Mina et al., 2013).

Significant progress has been made in deciphering key players of the transcriptional networks that control Fe homeostasis, and in understanding the genetic regulation of Fe uptake mechanisms in plants (Vert et al., 2003; Briat et al., 2007; Ivanov et al., 2012). The iron-regulated transporter (IRT1) and ferritin (FER) are proposed to be involved in iron transport, storage and protection against oxidative stress (Eide et al., 1996; Robinson et al., 1999). Genes transcriptionally regulated under conditions of low-Fe bioavailability synchronize iron uptake, translocation and storage in plant cells (Legay et al., 2012; Reyt et al., 2015). Iron-associated genes seem to play a vital role in the molecular responses to Fe deficiency-associated stress and tolerance in plants (Li et al., 2004; Ivanov et al., 2012; Legay et al., 2012; Darbani et al., 2013). Changes in the root transcriptome and morphology of Strategy I plants, for example, potatoes (Bienfait et al., 1987; Ivanov et al., 2012; Legay et al., 2012) in response to iron deprivation have been reported. Also, genes involved in iron acquisition are upregulated in response to Fe deficiency (Hindt and Guerinot, 2012; Kobayashi et al., 2012).

To reduce the agricultural and economic impact of iron deficiency stress, it has become necessary to develop an iron-efficient plant that is highly capable of optimum use of Fe for its metabolism. Such a plant can take up a smaller amount of Fe compared to a less efficient plant and produce the same yield (García-Mina et al., 2013; Vasconcelos and Grusak, 2014). Selection for Fe-efficiency in plants could enhance the use of marginalized calcareous soils while limiting Fe fertilizer usage and subsequently limit the economic losses associated with Fe deficiency. Biotechnological tools can be applied to develop plants that are adapted to Fe deficiency stress. *In vitro* plant cell selection is a cost-effective and practical tool for the selection of stress-tolerance in plants. The direct *in vitro* selection strategy prevents the development of epigenetically adapted cells (Chandler and Vasil, 1984; McHughen and Swartz, 1984; Tal, 1994). *In vitro* selection by indirectly inducing Fe deficit with incorporation of CaCO_3_ in tissue culture medium resulted in the generation of Fe-efficient lines of sugarcane plants (Naik et al., 1990). Palombi et al. (2007) employed *in vitro* regeneration to obtain and establish somaclones highly tolerant to calcareous soils. Two somaclonal variants recovered from shoots regenerated from quince leaves exposed to limited Fe supplies were found to be Fe-efficient (Dolcet-Sanjuan et al., 1992). Vasconcelos and Grusak (2014) identified Fe-efficient soybean plants and confirmed their tolerance to Fe-deficiency-induced chlorosis (IDC) under laboratory and field conditions. Comparative studies of iron-efficient (EF) and iron-inefficient (IFN) plants including investigations in relation to genes linked to iron homeostasis should lead to a better understanding of the molecular mechanisms governing stress responses to Fe-deficiency.

The generation of novel Fe-efficient potato variants *in vitro* is beneficial for crop improvement and can be used to complement conventional breeding. *In vitro* selection for Fe-efficiency trait in potatoes is valuable given the economic importance of potatoes and the prevalence of Fe-deficiency worldwide (Hindt and Guerinot, 2012; King and Slavin, 2013). However, there is a paucity of research to develop Fe-efficient potatoes based on *in vitro* plant cell selection. In this study, *in vitro* plant cell selection was applied to obtain EF and IFN *Solanum tuberosum* (cv “Iwa”) plant lines. The aim of this study was to gain insights from a comparison of the chlorosis symptoms and morphological characteristics of these novel plants and the parental biotype grown under *in vitro* and Fe deficiency conditions. Further insights were gained with an investigation into the transcriptional responses of the novel plant lines with differential tolerance to IDC. In particular, the expression of two key marker genes related to iron homeostasis, *irt1* and *fer3*, were investigated using RT-qPCR. It was hypothesized that the expression levels of *irt1* and *fer3* would be dissimilar in EF, IFN potato plant lines and the parental biotype.

## Materials and methods

### Plant material and growth conditions

Potato plantlets (*S. tuberosum* L. cultivar “Iwa”) were micropropagated using nodal explants and maintained on half-strength MS (Murashige and Skoog, 1962) medium as described previously (Yoon and Leung, 2004). All the media used in this study were supplemented with 3% sucrose (w/v) and the pH was adjusted to 5.7–5.8. For gelling the media 0.8% (w/v) agar (Oxoid, UK) was added and then autoclaved (20 min at 121°C) before the media were used. Cultures were maintained under controlled environmental conditions in a growth room at 22 (±2)°C with continuous lighting. Micropropagated potato plantlets were multiplied every 4 weeks by subculturing nodal explants onto fresh half-strength MS medium. The micropropagated potato biotype plantlets were used for the *in vitro* selection of potato variants for their tolerance to Fe-deficiency (see the overall experimental scheme in the Supplementary Table 1).

### Callus formation and selection of Fe-efficient callus cells

Young leaf explants were excised from the leaf stalk of micropropagated potato plantlets and cultured on half-strength MS medium supplemented with 3.22 μM of 1-napthalene acetic acid (NAA) and 1.78 μM of N_6_-benzylaminopurine (BA) in pre-sterilized plastic Petri dishes (9 cm diameter). After 4 weeks, the calli formed were transferred to fresh medium and allowed to proliferate during two subcultures (each of 4 weeks in duration). Next, a two-step direct selection strategy was employed with the sudden exposure of calli proliferated on Fe-sufficient medium (supplemented with 50 μM Fe) to Fe-deficiency conditions. The selection media against Fe-deficiency were supplemented with different concentrations of FeNa_2_EDTA (0, 0.001, 0.005, 0.01, 0.05, 0.1, 0.5, 1, 5 μM). Three pieces of excised calli (approximately 120 ± 10 mg) were cultured in each Petri dish. There were 30 biological replicates in a treatment and all experiments were repeated four times. Calli were subcultured every 4 weeks.

**Figure 1:**
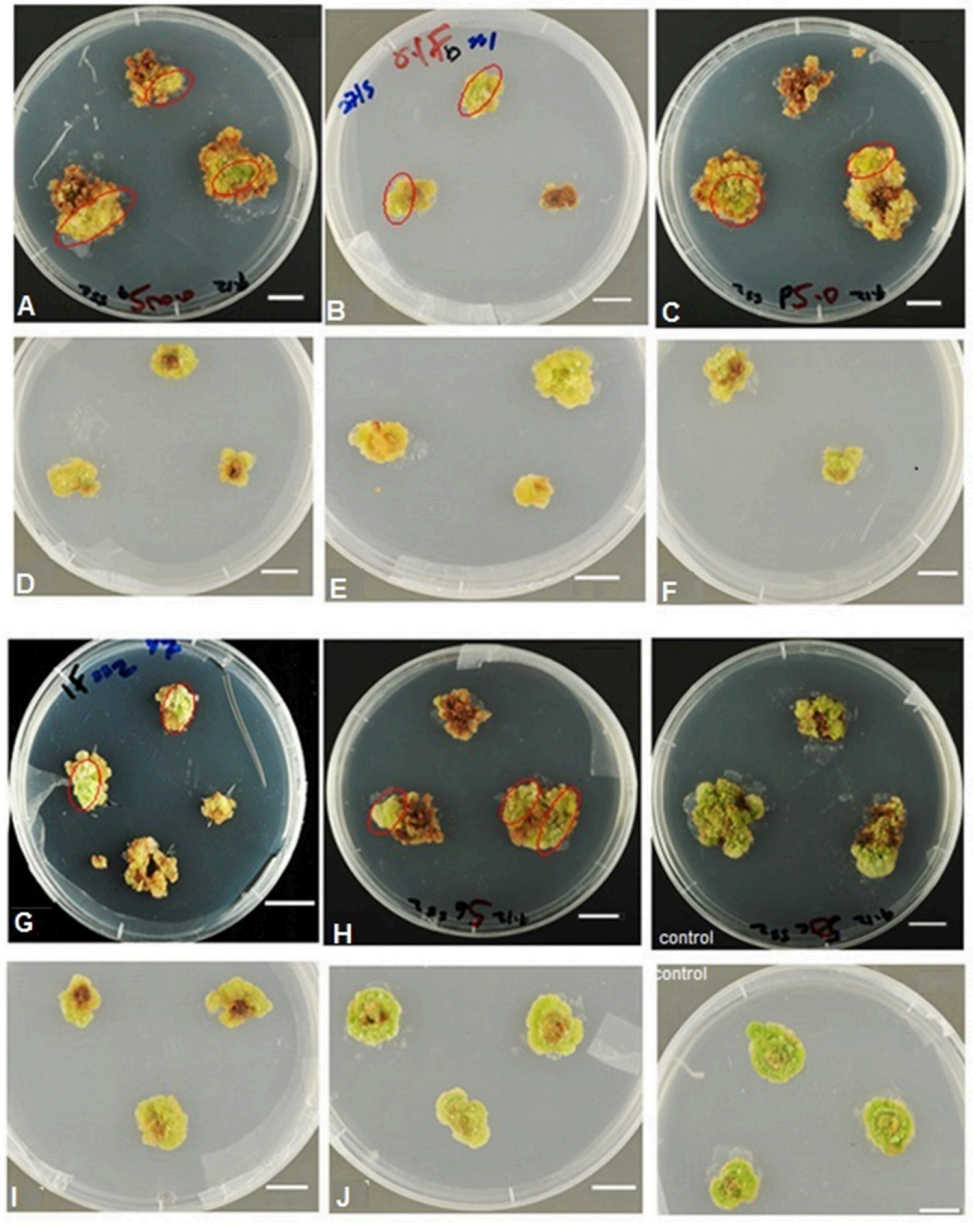
Selection for Fe-efficient potato (cv. “Iwa”) callus cultures using Fe deficiency as selection pressure. Isolated chlorosis-tolerant (“green islands”) somaclonal variants (circled in red) were subcultured on respective Fe-deficient medium for 2–4 subcultures to get rid of chimeras/mixed chlorosis-sensitive cells to obtain solely Fe-efficient callus lines. Calli were cultured on media supplemented with 0.005, 0.1, 0.5 **(A–C)**, 1 and 5 **(G,H)** μM Fe. Selected Fe-efficient calli subcultured on media supplemented with 0.005 – 0.5, 1 and 5 μM Fe **(D–F,I,J)**, respectively. Control: calli grown and subcultured on medium with sufficient Fe (50 μM). Scale of bars = 10 mm.

After 3 months of callus subcultures, somaclonal variants that seemed to be Fe efficient and apparently tolerant to Fe deficiency were selected as follows. Upon visual inspection, sections of green callus (“green islands,” Figure 1) free of surrounding dead cells were excised and placed onto freshly prepared selective Fe-deficient media. After three successive subcultures (each of 4 weeks in duration) on the Fe-deficient media, the calli that maintained proliferation ability and showed no chlorotic symptoms like the control (calli on Fe-sufficient medium) were denoted as chlorosis-tolerant (or Fe-efficient) calli (Figure 1).

### Plantlet regeneration and plant line establishment

**Figure 2:**
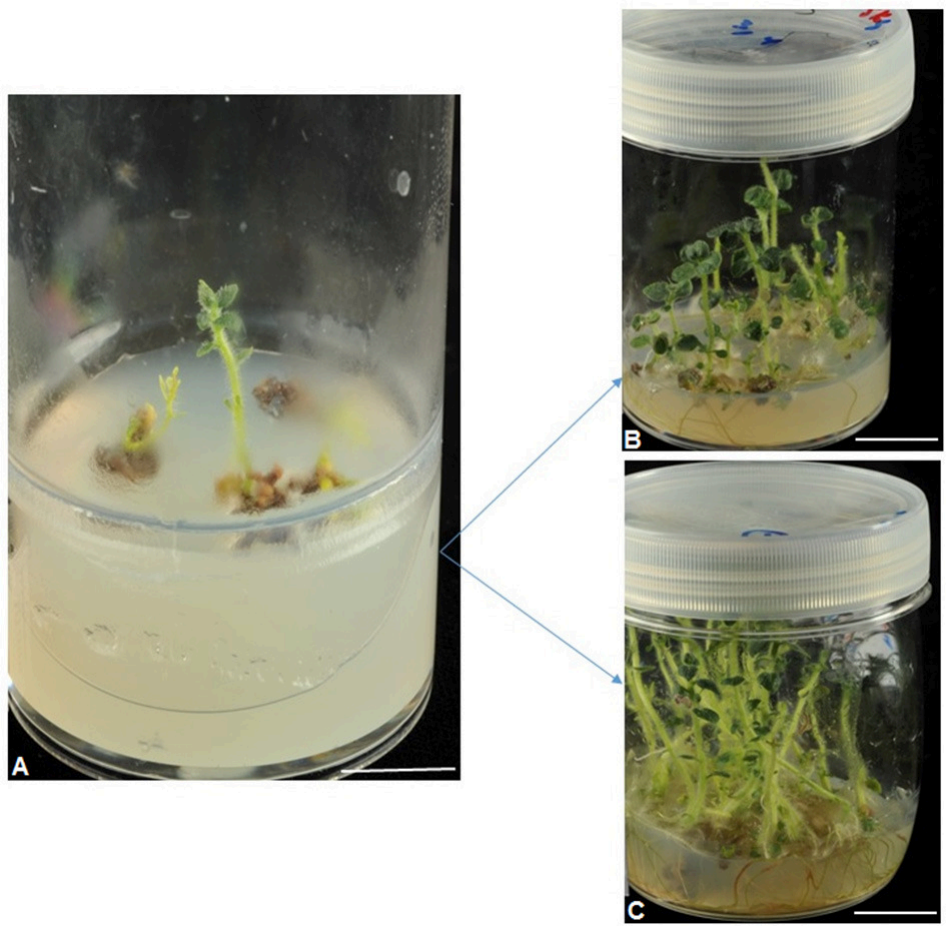
Establishment of selected plant lines, for example, derived from Fe-efficient potato (cv. “Iwa”) callus line selected when grown on medium supplemented with 0.005 μM Fe **(A)**. Two plant lines named as A1 and A2 (shown in **B** and **C**), respectively. Scale of bars: **A** = 10 mm, **B,C** = 20 mm.

To regenerate plants, the selected Fe-efficient calli were cultured on half-strength MS medium (containing 50 μM Fe) supplemented with 6.66 μM of BA and 2.89 μM of gibberellic acid (GA_3_). Pieces of calli which had developed shoot buds were transferred to half-strength MS medium devoid of plant growth regulators (PGRs) for plant propagation (Supplementary Figure 1). The shoots which did not elongate or proliferate well or with gross morphological aberrations were excluded from further experiments in this study. Individual adventitious shoots of 1.5 cm or taller were carefully excised from calli. These isolated shoots were subcultured 3–4 times (at 4-week intervals each) onto PGR-free medium for shoot elongation and rooting (Supplementary Figure 2). Regenerants with moderate-high growth rate and multiplication rates coupled with a high number of leaves produced were selected as putative Fe-efficient (EF) plant lines (Figure 2). Different plant lines were categorized based on the selection media from which the calli giving rise to the plant lines was selected (Figure 2). Shoots of the selected plant lines were micropropagated on half-strength MS medium to produce ample plant material for post regeneration screening of the selected plant lines under Fe-deficiency conditions.

### Post regeneration test for IDC tolerance

Plants of the selected lines and the parental biotype plants (SK) of similar size were subcultured three times over a 3-month period under *in vitro* Fe-deficiency conditions. For the screening and selection of Fe-efficient plant lines, there were 12 nodal explants or 12 biological replicates for each plant line and control plants. Plant lines were tested in media with stress levels corresponding to the Fe-deficiency selective pressure (0.005 to 5 μM Fe) under which Fe-efficient callus cells were originally selected. For example, plant lines derived from IDC tolerant callus cells selected on 0.005 μM Fe medium (A plant lines, Figure 2) were tested on medium with the same Fe content (0.005 μM). Similarly, plant lines B, C, D and E were tested on medium supplemented with 0.5, 0.1, 1 and 5 μM Fe, respectively.

Leaves were visually inspected for chlorotic symptoms by adopting the 1-5 points scale (Helms et al., 2010; Abadia et al., 2011; Vasconcelos and Grusak, 2014) for the identification of chlorosis tolerant plant lines. Based on the scale, visual chlorosis ratings (VCR) assigned were 1 = non-chlorotic (green leaf), 2 = minor/slight (pale green), 3 = average/moderate (moderate yellowing) and 4 = very chlorotic (intense yellow zones), 5 = extremely chlorotic (severe yellow to yellow-white or pink, bleached). All 35 plant lines were evaluated alongside the control plants in 3 repeat experiments. Chlorosis ratings of 12 replicates of each plant line were scored after 1 and 3 months of culture. Low VCR score (≤ 2.42) as a criterion for assessing chlorosis tolerance in the plant lines of this study.

### Plant growth measurements

The length, number, and color index of leaves of the plant lines were determined. Morphological parameters such as root, leaf and internode length as well as stem height were measured using a ruler. The length of leaves measured was the distance from the leaf apex to the base. The length of at least four leaves of a plant was measured. Total number of leaves and the distance between three internodes of a plant were counted and measured. There were at least eight replicates of each plant line and control plant for all parameters assessed.

### Real-time quantitative RT-PCR (qRT-PCR) analysis

Leaf and root samples harvested from potato plantlets were immediately frozen in liquid nitrogen and stored at –80°C. Two biological replicates each consisting of a pool of eight plantlets were used for RNA extraction. RNA extraction was carried out using the RNeasy Plant Mini Kit (Qiagen GmbH, Germany) according to the manufacturer's protocol incorporating an on-column treatment with RNase-free DNase I (Qiagen GmbH, Germany). The quantity and quality of RNA or DNA were assessed using a NanoDrop ND-1000 spectrophotometer V3.2 (BioLab Nanodrop Technologies, USA). The integrity of the isolated RNA was evaluated by running 1% agarose gel electrophoresis.

Complementary DNA (cDNA) was synthesized from 1 μg RNA by reverse transcription following the supplier's instructions for Transcriptor Reverse Transcriptase (Roche Diagnostics GmbH, Penzberg, Germany). RNA was reverse-transcribed with oligo-p(dT)_18_ primer (50 pmol) and random hexamer primer p(dN)_6_ (100 pmol) in the presence of 0.5 μl RNA secure (25x). Two separate reverse transcription assays were carried out for each sample.

The gene expression analysis was performed with KAPA SYBR® FAST qPCR Kits (Kapa Biosytems, Boston, USA) in a 72-well plate using Rotor-Gene Q (Qiagen). A reaction volume of 10 μl consisted of 5 μl of KAPA qPCR buffer, 1 μl of each forward and reverse primer, 2 μl of sterile Millipore® water and 1 μl of cDNA. Cycling conditions were 95°C for 10 min, followed by 40 cycles of 95°C for 10 s, 60°C for 15 s and 72°C for 20 s and with final extension at 72°C for 5 min. Gene-specific (*fer3, irt1*) and reference gene (cytoplasmic ribosomal protein, *L2*; elongation factor-1 alpha, *EF1*) qPCR primers used (Supplementary Table 2) were based on Legay et al. (2012) and Nicot et al. (2005).

The quality and yield of the cDNAs synthesized were assessed by performing qPCR on cDNAs of each sample using primers for two reference genes (*L2* and *EF1*) to evaluate the amplification curve and melting point curve. The assay was validated by optimizing primers to determine the ideal annealing temperature of all primers using 58, 60, and 62°C annealing temperatures. Identical replicates (*n* = 3) were used to monitor the accuracy of template and reagent pipetting, homogeneity of template and instrument performance. A minus reverse transcriptase (-RT) and a no cDNA template control (NTC) were included in every RT-qPCR run to test for gDNA contamination and to detect primer-dimers. The sample maximization strategy whereby all or as many samples as possible were analyzed for a given gene in the same run was followed. Since all samples could not be analyzed in the same run, an inter-run calibration (IRC) representing identical samples tested in every run was performed (Hellemans et al., 2007). The IRCs served as positive template controls (cDNAs pooled from all samples) and were generally included to check for consistency of reaction.

### Data analysis

IBM SPSS (version 23) software was used for statistical analysis. For morphological parameters, the mean length of stem, leaf, root, internode, and the number of leaves per plant were the variables. Correlation and binary logistic regression analyses were done to determine the relationship between variables and the associations between exposure to Fe deficiency and outcomes. Relative quantification of *fer3* and *irt1* expression levels was calculated taking two reference genes (*EF1* and *L2*) into account based on the calculation, ΔCq (expression = 2^−ΔCq^), and the method described by of Song et al. (2012). The expression of the two reference genes was adjusted by calculating a correction factor (CF) for individual cDNAs for each reference gene using the Cq values. The final CF value was calculated by averaging the CF values (four replicates per cDNA sample) of the two reference genes and its correlation to the target genes *fer3* and *irt1* was evaluated. The Cq values of each target gene was corrected before data analysis. Normalized relative expression data are means of relative mRNA levels in fold changes detected using four replicates for each biological replicate (*n* = 8). Significant differences between samples were calculated using one-way ANOVA, followed by Least-significant difference (LSD) and Duncan's multiple range tests after checking the data normality and homogeneity of variances at 0.05 significance level. *T*-test was performed to evaluate statistical differences in mean expression levels of the root and leaf.

## Results

### Selection of Fe-efficient calli

A majority of potato calli (60–80%) appeared chlorotic (light yellow), or bleached and even necrotic after 3 months of culture on medium supplemented with 0 to 5 μM Fe compared with the control (50 μM Fe) (Figure 1). However, some calli cultured on Fe-deficient medium (0 to 5 μM Fe) survived and increased in growth area even after 3 months of culture. Sections of few of the calli cultured on media supplemented with 0.005–5 μM Fe maintained green pigmentation (“green islands,” Figure 1) and appeared comparable to control calli. These were potential Fe-efficient somaclonal variants and were selected for further studies and plant regeneration. Approximately 56% of the potential Fe-efficient callus lines regenerated into plants which were categorized as different plant lines, A–E, as described in the Materials and Methods.

### Post regeneration test for IDC tolerance

**Figure 3:**
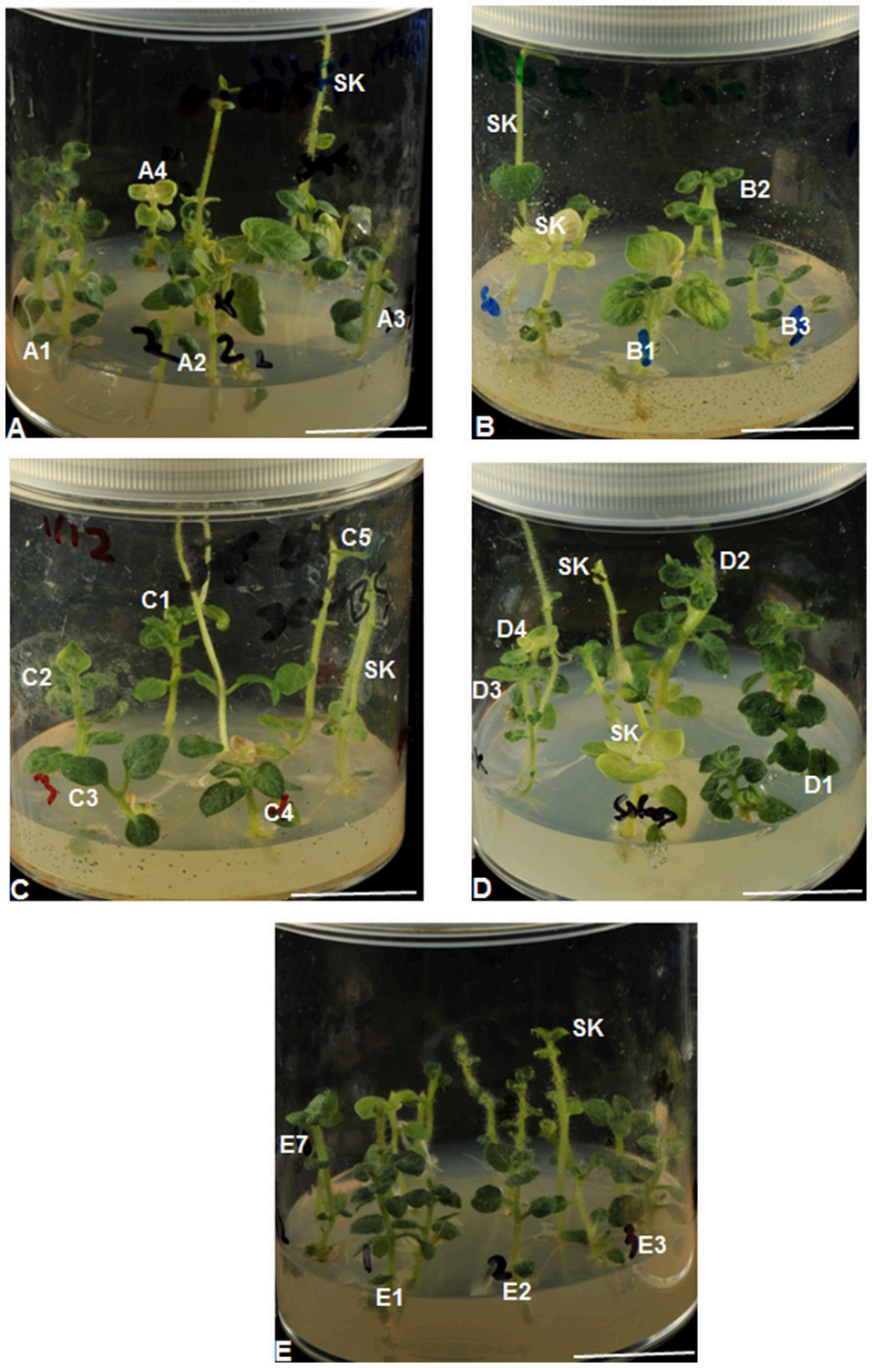
Differential tolerance to Fe deficiency-induced chlorosis among potato (cv. “Iwa”) plant lines and control plants (SK). Top to bottom: **(A–E)** plant lines, that were regenerated from calli tolerant to low Fe levels (0.005–5 μM Fe), were grown on the same respective medium (**A**: 0.005 – **E**: 5 μM Fe) used for isolation of the calli. Plants are indicated as Fe-efficient (A1, B2, D1, E1–3, and E7) and Fe-inefficient (A2–4, B1, B3, C1–5, D2–4, E6, and SK). All scale bars = 20 mm.

**Figure 4:**
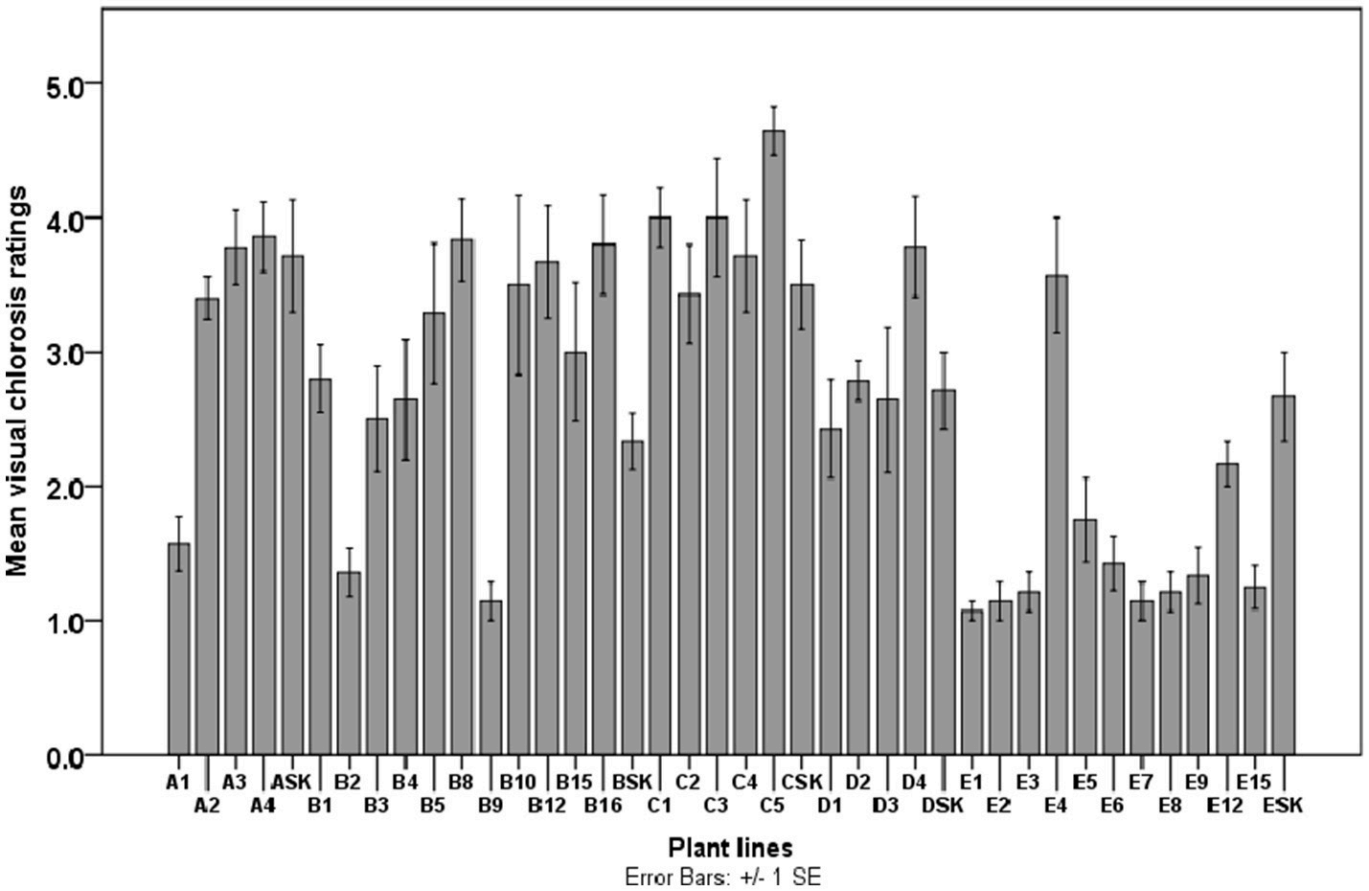
Visual chlorosis rating scores of potato plant lines (A–E) and parental biotype (SK) cultured in Fe-deficient medium supplemented with 0.005 μM Fe (for A1-ASK plant lines derived from Fe-efficient calli selected on 0.005 μM Fe-containing medium), 0.5 μM Fe (for B1-BSK), 0.1 μM Fe (for C1-CSK), 1 μM Fe (for D1-DSK) and 5 μM Fe (for E1-ESK). Chlorosis scores were taken within 3 months of culture for all 12 biological replicates per plant line and control plant.

There was a clear distinction between IDC sensitive (severely chlorotic) and tolerant (non-chlorotic) plantlets after 3 months of exposure to Fe-deficient medium (Figures 3, 4). The severity of chlorosis appeared to be plant line-specific and independent of the extent or magnitude of Fe deficiency in the medium. Eight (23%) Fe-efficient (EF) and 27 (77%) Fe-inefficient (INF) plant lines were identified (Figures 3, 4). The parent biotype (SK control) plants were chlorosis sensitive. The selected EF plant lines had a low combined average chlorosis score of 1.4 ± 0.4 (SD) and the INF lines ones had a high score of 3.5 ± 0.6 (SD). The potato lines selected for increased tolerance to IDC were A1, B2, B9, D1, E1-3 and E7. Consistently, the A1 plant line showed a lower degree of leaf chlorosis within 3 months of exposure to 0.005 μM Fe medium compared to other A plant lines cultured in the same medium. Like A1, lines B2, B9, E1–3, and E7 seemed to exhibit considerable tolerance to Fe deficiency. All the C lines had mean chlorosis scores greater than three and were classified as IFN plants. Line D1 did not exhibit chlorotic symptoms in the initial 4 weeks of culture on 1 μM Fe medium and had the least mean VCR value (2.40) among the D plant lines after 3 months. Although plant lines E5, E6, E8, E9, and E15 had considerably low IDC scores (1.20 to 1.60), they were not considered as promising EF plant lines because they exhibited atypical morphological features such as thin short shoots, fibrous-like stems, with tiny leaves or lacking any leaf development. Moreover, their growth rates were considerably slower under Fe-deficiency conditions.

### Plant growth measurements

**Figure 5:**
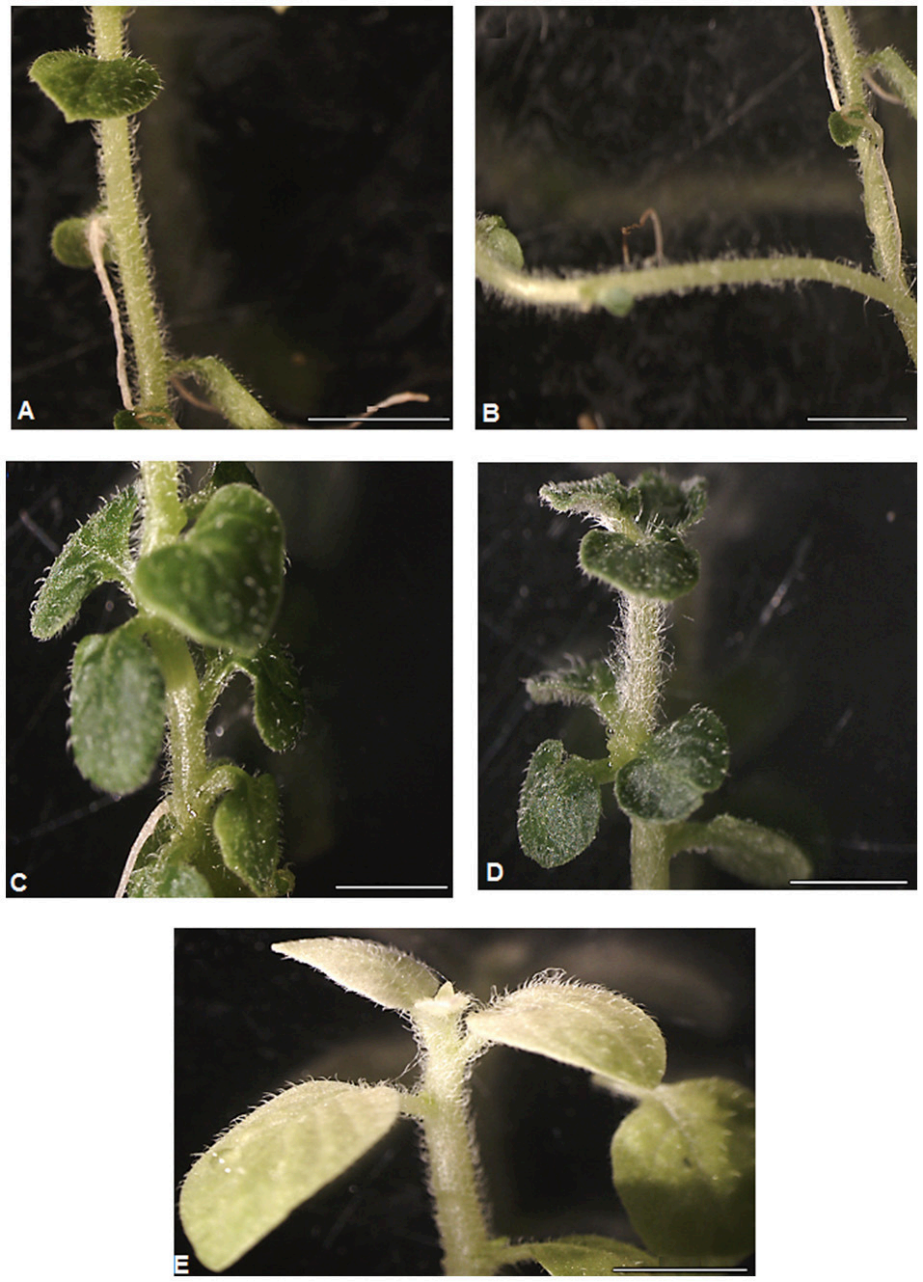
Stereomicroscopic view of morphological characteristics of parental potato (cv. “Iwa”) stock plants **(A,B)**, and plant lines **(C–E)** growing under Fe deficiency conditions. From top to bottom: control plants **(A,B)** with slender to thin long stems, lateral stem, long intermodal distance, and fewer number of leaves; Fe-efficient **(C,D)** and inefficient **(E)** plant lines with thick short stems, short intermodal distance, numerous average to broad sized and chlorotic leaves **(E)**. Scale of bars = 5 mm.

**Figure 6:**
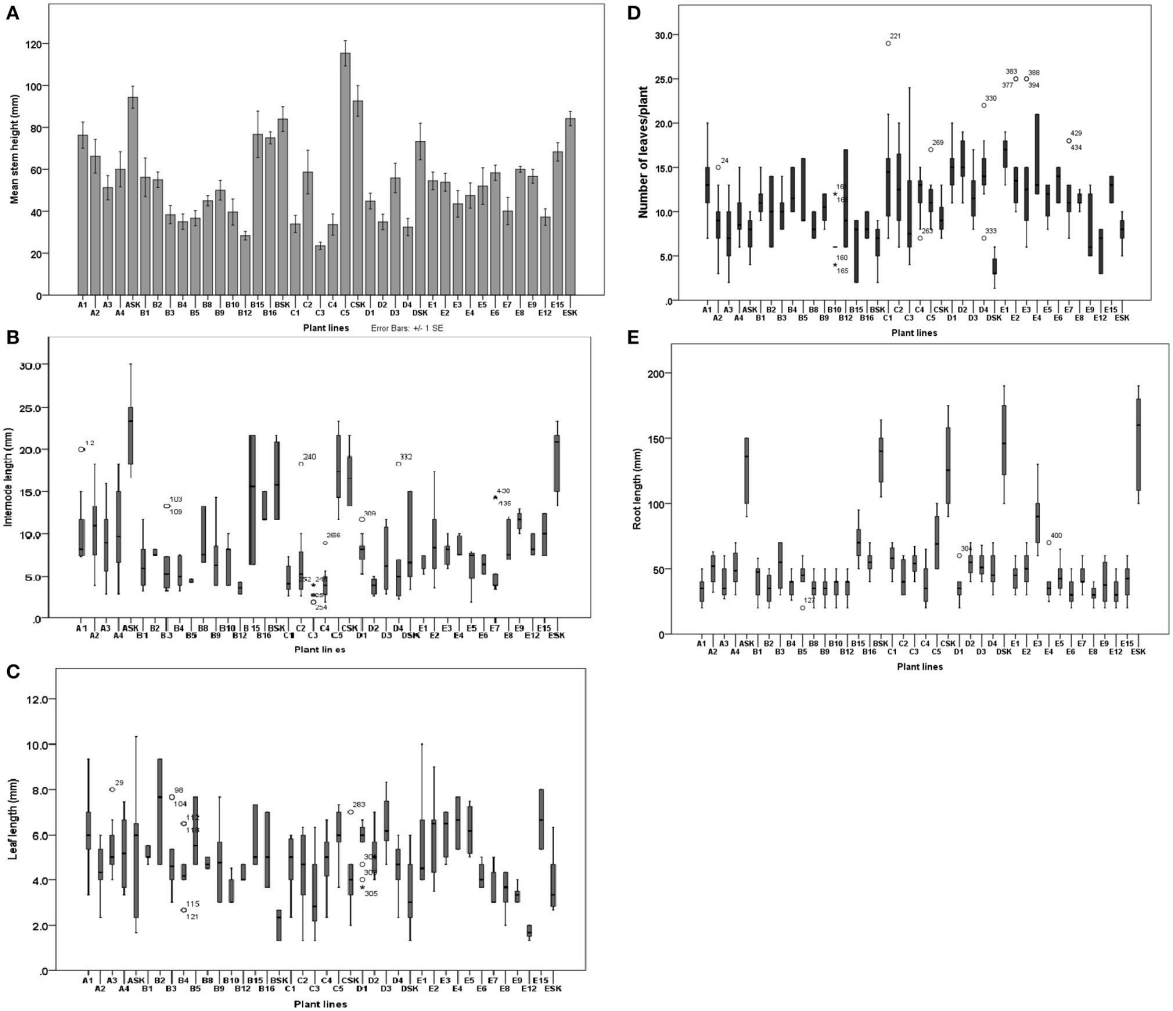
Effect of Fe deficiency on stem height **(A)**, intermodal distance **(B)**, leaf length **(C)**, number of leaves per plant **(D)** and root length **(E)** of potato lines and control (cv. “Iwa”) parental biotype (SK). Morphological parameters were measured (*n* = 9) after 2 months of exposure to Fe deficiency treatments (0.005 μM Fe for A plant lines, 0.5 μM Fe for B lines, 0.1 μM Fe for C lines, 1 μM Fe for D lines and 5 μM Fe for E lines).

The morphology of plant lines and the control parental biotype were assessed in order to study the influence of Fe-deficiency stress conditions on growth of the stem, leaf and root. Under Fe deficiency conditions, the control plants were characterized by tall, thin and bending shoots, greater internodal distances, small to medium-sized leaves (Figures 5A,B) and high sensitivity to chlorosis. Contrarily, most EF and INF plant lines had relatively shorter and thicker stems with shorter internodes. Most EF lines had greener leaves than control (Figures 5C,D) while IFN lines had yellow chlorotic leaves (Figure 5E). Control plants had a greater propensity to develop lateral stems compared to other plant lines (Figure 5B). Stem heights of plant lines ranged from 23.5 ± 6.15 (in C3) to 76.3 ± 22.6 cm (in A1) while those of the controls ranged from 72.4 ± 34.1 to 92.6 ± 20.7 cm (Figure 6A). Unlike most EF lines, the stem of A1 line was almost as tall as the control plants (Figure 6A). The internodal distances of control plants were 2 to 7 times greater than those of the plant lines (Figure 6B). Leaves of the parental biotype, SK, had significantly reduced leaf size and length (Figure 6C) as well as decreased number of leaves per plant (Figure 6D) compared to the plant lines. Roots of the control plants were significantly longer than IFN and EF plant lines by 2 to 5 times (Figure 6E). Control plant roots were thin and green in color with few or no lateral roots compared to the plant lines (Supplementary Figure 3). The length, thickness and density of root hairs formed in the plant lines appeared to be greater than those developed in control plants (data not shown). Most EF plant lines had reduced root lengths with a greater number of lateral roots than IFN lines. Some IFN plant lines (e.g., B15, B16, and C5) showed either enhanced or similar morphological characteristics to that of the control plants (Figure 5): they had slender or thin elongated shoots with long intermodal distances, long roots and were severely chlorotic.

### Correlations and associations among morphological characteristics of plant lines

The number of leaves in a plant was negatively correlated with internodal distance but positively related to leaf length (Table 1). The plant lines with shorter internodal distances had a greater number of leaves and longer leaves. Most EF plant lines and some IFN lines displayed the aforementioned characteristics. There was a weak correlation of visual chlorosis scores (VCRs) with stem height, number of leaves, leaf and internode lengths. However, VCR was strongly related to root length (Table 1). This suggests that IDC sensitivity (in control and IFN plants lines) may be connected to modifications in plant stature and architecture but to a lower extent in the shoots than the roots. Plant root length appeared to contribute to IDC susceptibility since there was a significantly positive correlation between VCR score and root length. Enhancement in root length was significantly related to increase in stem height. This was characteristic of the control plants under Fe deficiency conditions. Stem height had a highly positive relationship with intermodal distance, leaf and root lengths. This implies that IFN and control plants that were taller had longer roots, leaf length and intermodal distances as summarized in Table 1.

**Table 1:** Spearman's correlation coefficients for visual chlorosis scores and morphological parameters measured in potato plant lines and control plant (cv. “Iwa”).

**Parameter**	**Stem height**	**Internode length**	**Leaf length**	**Number of leaves/plant**	**Root length**	**Visual chlorosis score**
Stem height	1.00	0.71^**^	0.17^**^	−0.04	0.35^**^	−0.03
Internode length	0.71^**^	1.00	0.09	−0.14^**^	0.30^**^	0.01
Leaf length	0.17^**^	0.00	1.00	0.25^**^	0.07	0.06
Number of leaves/plant	−0.04	−0.14^**^	0.25^**^	1.00	−0.07	−0.11
Root length	0.35^**^	0.30^**^	0.07	−0.07	1.00	0.30^**^
Visual chlorosis score	−0.03	0.01	0.06	−0.11	0.30^**^	1.00

Plant materials were collected and assessed after 2-month exposure to Fe-deficiency conditions.

** *Correlation is significant at the 0.01 level*.

There was a significantly positive association between control plants and the likelihood of having a high chlorosis score (chlorosis sensitive) with increased stem height, root and internodal length upon exposure to Fe-deficiency conditions (Table 2). A unit increase in chlorosis score, stem height, root, and internode length raises the likelihood of an association with the parental biotype, SK. Odds ratio for leaf length, number of leaves per plant was less than 1 (Table 2) suggesting that these characteristics had a low likelihood of being associated with control plants exposed to low Fe-containing medium. It can be inferred that such characteristics could be associated with the selected potato plant lines. There was a strong link between control plants and IFN plant lines and the likelihood of susceptibility to chlorosis.

**Table 2:** Odds ratio, Exp(B), values for the association of morphological characteristics of potato (cv. “Iwa”) plants and exposure to Fe-deficiency.

**Morphological parameters**	**Exp(B)**	**Significance**
Chlorosis	1.70	0.00
Root length	1.13	0.00
Number of leaves/plant	0.90	0.00
Leaf length	0.97	0.59
Internode length	1.26	0.00
Stem height	1.04	0.00

*Control plants and plant lines were cultured in Fe-deficient medium for 2 months*.

### Enhanced *fer3* expression associated with Fe-efficiency

**Figure 7:**
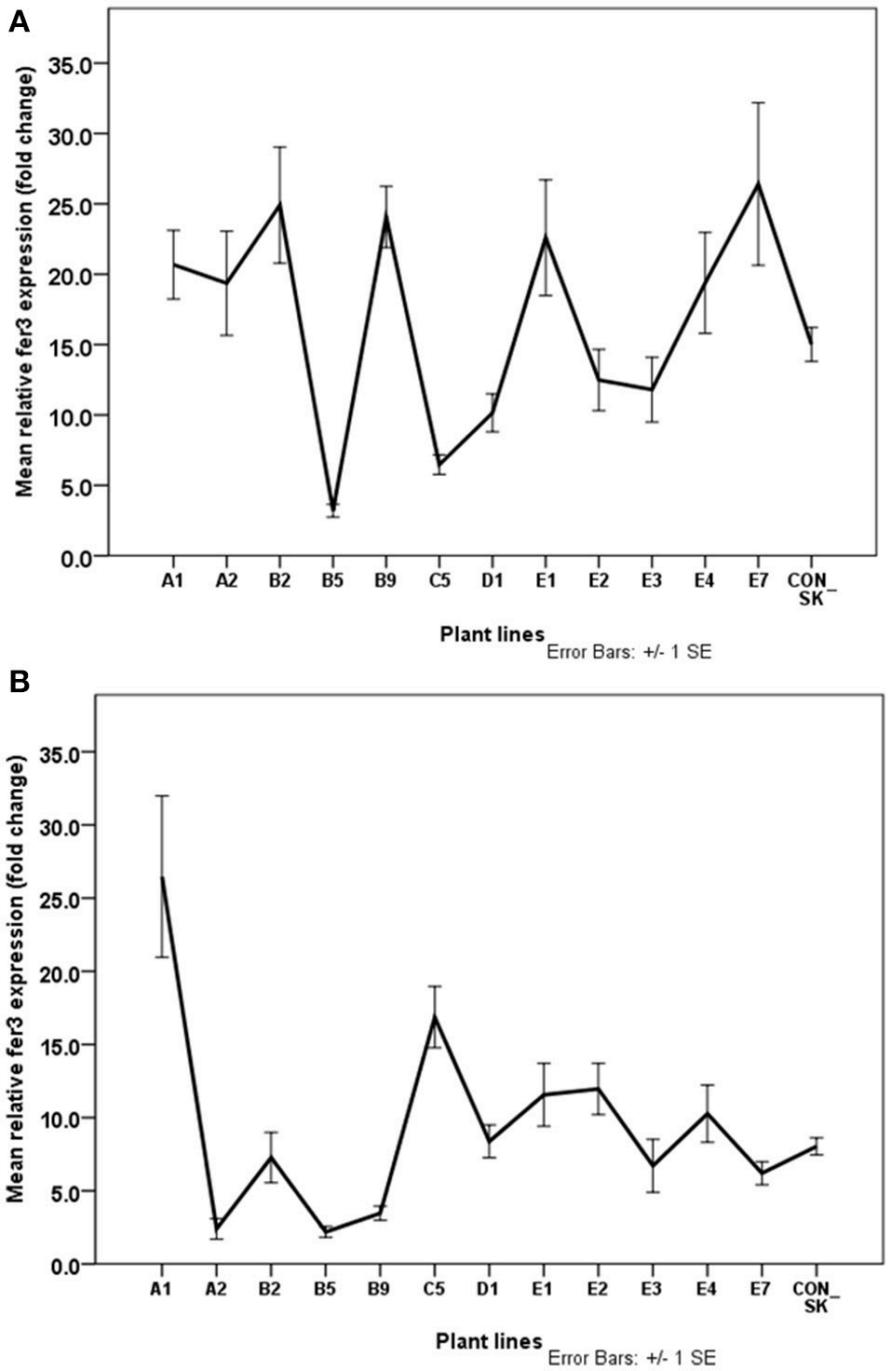
Normalized relative expression of *fer3* in leaf **(A)** and root **(B)** of *S. tuberosum* (cv. “Iwa”) plants with differential tolerance to Fe-deficiency induced chlorosis. Plants were exposed to Fe-deficiency conditions for three months. Values are means of relative transcript levels (in fold change) of four replicates for each of two biological replicates (*n* = 8). *EF1* and *L2* reference genes were used to normalize *fer3* expression levels. Error bars represent the ± standard error (SE) of the mean calculated for the combined sample and biological replicates. A1, B2, B9, D1, E1-3 and E7 represent plant lines selected to be potentially Fe-efficient based on IDC scores ≤2.42.

Approximately 62% of the Fe-efficient plant lines including A1, B2, B9, E1, and E7 had significantly increased *fer3* expression levels in the leaf compared to the parental biotype plants under Fe-deficiency conditions (Figure 7A). The lowest *fer3* expression was detected in the leaves of the IFN lines, C5 and B5. Expression of the *fer3* gene was elevated in the roots of 37.5% of the potential Fe-efficient plant lines (namely, A1, E2, and E1) compared to the parental biotype (con-SK) (Figure 7B). Expression of *fer3* was highest in the root of the A1 plants and was 53% higher than that in the con-SK plants. Overall, in both the leaves and roots of A1 and E1 plants, *fer3* expression was significantly higher than the parental biotype (con-SK). In 50% of the Fe-efficient lines (D1, B2, E7, and E3), *fer3* expression (6–8 fold change) in the root was similar (*p* > 0.05) to that of con-SK. Interestingly, C5 (the Fe-inefficient line, although severe chlorotic (Figure 4), showed elevated *fer3* expression in the root compared to the control plants and other EF plant lines except A1.

### Fe-deficiency induced changes in *irt1* expression

**Figure 8:**
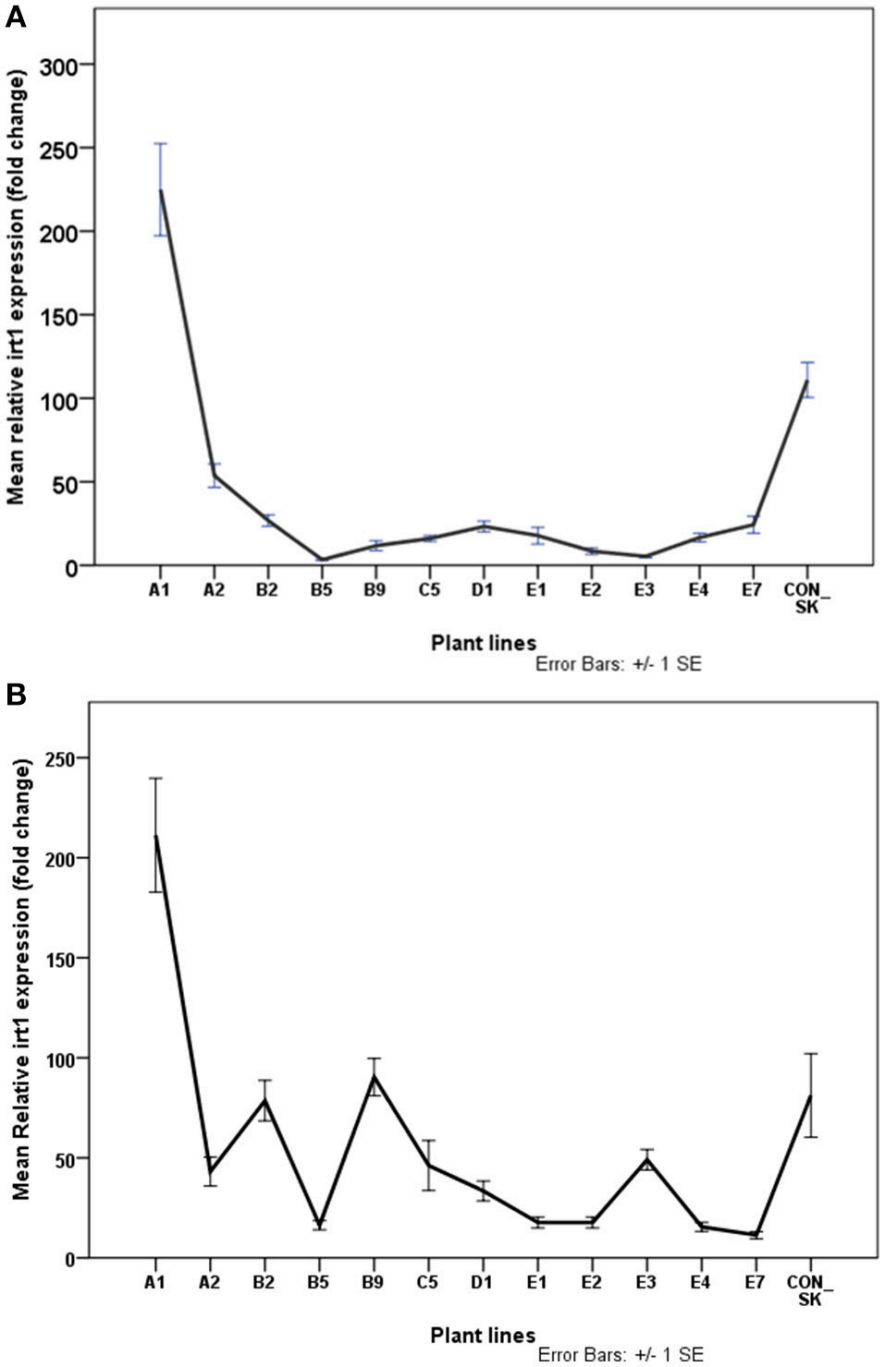
Normalized relative expression of *irt1* in leaf **(A)** and root **(B)** of *S. tuberosum* (cv. “Iwa”) plants with differential IDC tolerance. Plants were exposed to Fe-deficiency conditions for three months. Values are means of relative transcript levels (in fold change) of four replicates for each of two biological replicates (*n* = 8). *EF1* and *L2* reference genes were used to normalize *irt1* expression levels. Error bars represent the ± standard error (SE) of the mean calculated for the combined sample and biological replicates. A1, B2, B9, D1, E1–3, and E7 are plant lines selected to be potentially Fe-efficient based on IDC scores ≤2.42.

Expression of *irt1* was generally significantly higher by 20% (*t* = −3.24, *df* = 222, *p* < 0.05) in roots than in leaves in most plant lines studied except A1. Expression of *irt1* in the leaf of A1 (224 fold change) was comparable to that in the root of A1 (211 fold change) and both values were significantly higher than those in the control plants (Figure 8). The increase in *irt1* expression in the leaf of A1 plants was twice as much as that of the parental biotype (*p* < 0.05). In contrast, the expression of *irt1* in the leaves of about 88% of the putative Fe-efficient lines and all the IFN plant lines studied was lower than in the parental biotype (Figure 8A). The root of A1 plants exhibited the highest expression of *irt1* which was significantly higher (by 44%) than the root of the parental biotype (Figure 8B). In all IFN plant lines, *irt1* expression in the root was lower than the control plants (*p* < 0.05). Surprisingly, the roots of some EF plant lines (E1, E2, and E3) had the lowest *irt1* expression levels.

## Discussion

Fe^2+^ deficiency can restrict plant growth and yield. Development of Fe-efficient plant varieties is suggested to be the best practical means to correct IDC and avert plant growth restrictions due to Fe^2+^ deficiency in soils (Hansen et al., 2006; Helms et al., 2010; García-Mina et al., 2013; Vasconcelos and Grusak, 2014; Zhu et al., 2015). This is the first report on *in vitro* selection of Fe-efficient potato plant lines. In the present study, low concentrations of Fe in culture medium were used as selective pressures. This direct selection scheme was designed to recover the preferential survival of somaclonal variants adapted and tolerant to the Fe-deficiency stress applied. Therefore, the advantage of the present approach over the use of CaCO_3_ as a selection agent in other previous studies was to avoid uncovering variations related only to CaCO_3_ *per se* (Naik et al., 1990; Dolcet-Sanjuan et al., 1992; Palombi et al., 2007; Kabir et al., 2015).

The initial selection by visual screening enabled the identification of IDC-tolerant callus lines over sensitive ones based on differences in pigmentation and cell survival. When cells experience trauma or stress, they may reset genome expression (Phillips et al., 1994). Such reprogramming can give rise to somaclones with altered gene expression in plants arising from point mutations, gene amplification, chromosomal rearrangements and recombination, DNA methylation, ploidy change, altered sequence copy number, activation of transposable elements, and chromatin loss (Phillips et al., 1990; Rao et al., 1992; Kaeppler et al., 2000; Kumar and Mathur, 2004; Pérez-Clemente and Gómez-Cadenas, 2012).

The putative Fe-efficient potato plant lines obtained from selected callus cultures were subjected to Fe-deficiency conditions *in vitro*. In this way, the stability of the trait was determined so that the most promising Fe-efficient plant lines could be selected for further plant germplasm evaluation and breeding. The main advantage of testing the plant lines under Fe-deficiency conditions was that IDC-tolerant plant lines could be identified and distinguished from IDC-susceptible plants. Similar to the study of Vasconcelos and Grusak (2014), plants with an average VCR of ≤2.42 or ≥3.50 were designated as chlorosis-tolerant (i.e., Fe-efficient: EF) or chlorosis-sensitive (Fe-inefficient: INF) lines, respectively. Here, the chlorosis tolerance trait was found to persist in the selected EF plant lines over long-term subculturing under Fe-deficiency stress conditions. The selected Fe-efficient plant lines had mild to no symptoms of chlorosis and grew better on Fe-deficient medium than chlorosis-susceptible plants (IFN and controls). Likewise, in *M. ciliaris*, Fe deficiency had a more pronounced impact (severe chlorosis) on IDC-sensitive lines than tolerant ones (M'sehli et al., 2014).

The morphological characteristics of the Fe-efficient potato plants obtained were similar to those observed in other studies. For example, Fe-efficient soybean plants were observed to be shorter compared to inefficient ones (Elmstrom and Howard, 1969; Vasconcelos and Grusak, 2014). Similarly, the stem height of both chlorosis-sensitive and tolerant chickpea varieties exposed to Fe deficiency was markedly lower than the respective controls (Mahmoudi et al., 2009). Under Fe deficiency conditions, shoot height, fresh weight and growth were reduced significantly in chlorotic compared to non-chlorotic plants (M'sehli et al., 2008; Kabir et al., 2015). Morphological variations in leaf development and size have been linked to Fe deficiency chlorosis. Chlorotic leaves exhibited significantly reduced leaf expansion, size, fresh, and dry weight (Larbi et al., 2006; Fernández et al., 2008) compared to non-chlorotic leaves under Fe-sufficient conditions (Maldonado-Torres et al., 2006). Fernández et al. (2008) proposed that IDC can prevent leaf development via changes in leaf cuticle and cell wall. They showed that green leaves had thick and homogeneous cell walls while cells in chlorotic leaves appeared as thin, discontinuous and heterogeneous. Fe-deficiency can modify the barrier properties of the leaf surface, which can have a marked effect on leaf water relations, solute permeability and pest and disease resistance (Fernández et al., 2008). Fe shortage results in reduced leaf growth, leaf number, leaf surface area, leaf biomass, loss of turgor and the inhibition of the formation of new leaves (Kosegarten and Koyro, 2001; Mahmoudi et al., 2009; Kabir et al., 2015). Poor leaf growth and suppressed leaf formation are characteristic symptoms of IDC response due to the high sensitivity of the meristematic apex to low iron availability (Gruber and Kosegarten, 2002; Vasconcelos and Grusak, 2014).

In strategy 1 plants (e.g., potato), Fe-deficiency is associated with inhibition of root elongation, increase in diameter of apical roots, subapical swelling with abundant root hairs and formation of transfer cells (Bienfait et al., 1987; Broadley et al., 2012). The most efficient way of increasing absorbing root surface area is in the formation of root hairs. In the current study, the increased number of lateral roots and root hairs identified in the potato plant lines especially the Fe-efficient ones are proposed to be necessary for a more efficient exploration of the medium for the acquisition of Fe. Also, most EF plant lines had reduced root lengths than IFN lines. This is contrary to findings in previous reports on other plants which were evaluated under different conditions. Increase and decrease in root biomass was observed in *M. ciliaris* chlorosis tolerant and sensitive ecotypes, respectively (M'sehli et al., 2008). Similarly, a significant decline in root length and fresh weight was observed in a chlorosis-susceptible okra variety compared to a tolerant variety under low Fe availability (Kabir et al., 2015). Although root biomass was unaffected by Fe in medium, reduction in dry weight and root biomass was more severe in chlorosis-susceptible chickpea varieties than tolerant ones in comparison to their respective controls under bicarbonate-induced Fe deficiency (Mahmoudi et al., 2009). Fe-deficient plants developed more lateral roots and abundant root hairs (Licciardello et al., 2013). Improvement in root hair formation in Arabidopsis mutant was regulated by the Fe concentration in the growth medium (Schikora and Schmidt, 2001). In the present study, the Fe-efficient plant lines that exhibited increased lateral roots, root hairs and decreased length could be of interest for further evaluation in the field. In tomato (Zamboni et al., 2012) and potato (Bienfait et al., 1987), enhanced root hair proliferation is a morphological adaptation linked to suboptimal Fe conditions. When plants growing under Fe deficient conditions were resupplied with Fe, morphological adaptations exhibited by the root were diminished and transfer cells became degenerated (Broadley et al., 2012).

Plant ferritin plays an essential role in the maintenance of iron buffering, oxidative stress prevention and adaptation to adverse environmental situations (Ravet et al., 2009; Briat et al., 2010; Parveen et al., 2016). According to Zok et al. (2010), increased production of ferritin improved abiotic stress tolerance in transgenic grapevine plants. In response to excess Fe supply there was an up-regulation of ferritin (*fer1*) transcript in chickpea seedlings (Parveen et al., 2016) and *fer3* in potato leaves (Legay et al., 2012). In transgenic chickpea plants, overexpression of ferritin (*fer1*) was found to confer enhanced growth (Parveen et al., 2016). An interesting finding from the present *in vitro* cell line selection study is that the expression of *fer3* was significantly higher in the leaves of predominantly IDC-tolerant potato plant lines compared to IDC-sensitive plants under Fe deficient conditions. A plausible explanation for this result could be that the induction of *fer3* expression due to low Fe availability possibly serves as a response mechanism to improve Fe acquisition in IDC- tolerant plants. The tissue culture-derived variant IDC-tolerant plants might have this as an adaptive mechanism to enable survival under Fe-deficiency stress conditions. This remains to be elucidated further, however. Ferritin and Fe are co-localized primarily in chloroplasts of leaves storing Fe where photosynthesis is active (Briat et al., 2006, 2010; Darbani et al., 2013). It is not surprising that more pronounced ferritin expression was found in leaves than in roots. Elevated *fer3* expression in the putative Fe-efficient lines may be connected to reduced chlorosis observed in EF lines which had high leaf chlorophyll contents (Boamponsem, 2017).

In the root, the functional role of ferritin or its involvement in oxidative stress responses has been studied less thoroughly and some findings are debatable (Vigani et al., 2013; Reyt et al., 2015). Santos et al. (2015) have reported that Fe-inefficient plants (at an early growth stage) had higher induction levels of the *ferritin* gene in the roots than Fe-efficient plants. Vigani et al. (2013) reported that ferritin was abundant in mitochondria of the roots of cucumber plants grown under Fe-excess. However, lower ferritin levels were detected in roots of the cucumber plants grown in medium with sufficient Fe. Reyt et al. (2015) also reported that the ferritin gene, *fer1*, was most highly expressed in Arabidopsis roots in response to excess Fe. Low-iron availability led to the repression of *fer3* expression in the roots of potato plantlets whereas in high Fe medium, *fer3* expression was significantly higher (Legay et al., 2012). In the present study, the level of *fer3* expression was strongly increased in the roots of some of the Fe-efficient and Fe-inefficient lines and control plants. However, *fer3* expression was markedly reduced in other lines. It appears, therefore, that differential tolerance to IDC in potato plants may not be linked to *fer3* expression levels in the root.

The literature on *irt1*expression in leaves is scanty. Earlier studies by Vert et al. (2002) have shown that *irt1* expression is noticeable in leaves and not restricted to the roots of Arabidopsis. The expression of *irt1* gene was detected in the rosette leaves and found to be expressed in the basal part of flowers, suggesting its role in Fe acquisition in aerial tissues. Here, *irt1* expression in leaves was generally low, but significantly high levels were identified in the A1 line, the A2 Fe-inefficient line and the parental control plant. Similarly, Santos et al. (2015) indicated that IRT1-like gene expression was high in leaves of both EF and IFN soybean plants under Fe-liming conditions. Contrarily, in examining plants' response to Fe supply, it was observed that *irt1* mRNA was reduced in leaves of plants grown in iron-limited conditions (Barberon et al., 2011; Legay et al., 2012).

Vert et al. (2002) observed severe leaf chlorosis typical of iron-deficient plants in the Arabidopsis knockout mutant (*irt1-1*). In the present work, not enough evidence has been found to imply that high chlorophyll content in leaves of potential Fe-efficient potato lines may be directly related to the high expression of *irt1* in leaves. This pinpoints a possible lack of correlation between visual chlorosis symptoms and the molecular mechanisms associated with leaf iron transport. Legay et al. (2012) found a strong increase in *irt1* expression in roots of potato plants grown in low Fe-containing medium. In contrast, *irt1* expression was decreased when the potato plants were grown under high Fe excess. In the present study, expression levels of *irt1* were significantly higher in roots than in leaves. Fe deficiency induced changes in *irt1* transcript levels largely in roots of potato plant lines with differential tolerance to Fe-deficiency. This is consistent with the notion that *irt1* has a role in Fe uptake in potatoes (Figure 8).

In the roots of 50% of the potential Fe-efficient plant lines derived from *in vitro* selection here, *irt1* expression was enhanced as a consequence of Fe deficiency (Figure 8). Additionally, *irt1* was expressed at increased levels in the roots of both IDC-tolerant and -sensitive potato plant lines with a more pronounced effect detected in the IDC-tolerant line, A1. These findings are consistent with earlier reports on *irt1* expression in okra plants with differential IDC tolerance (Kabir et al., 2015). *Irt1* seems to play a vital role in the response of potato plants exposed to Fe-deficiency conditions by enhancing Fe-transport capacity, mainly in the roots in agreement with previous reports (Vert et al., 2002; Legay et al., 2012; Kabir et al., 2015).

There have been conflicting reports and divergent conclusions concerning the molecular responses of EF and IFN plants under Fe limiting conditions. Some studies have shown that the expression of genes associated with Fe-deficiency was increased in EF plants (Kabir et al., 2015). However, IFN plants have also been found to exhibit enhanced expression of these genes (Martínez-Cuenca et al., 2013; Santos et al., 2015). In the present study, it was also not possible to establish specifically that Fe-efficiency might be attributable to higher *irt1* expression in the root as about the same number of EF lines exhibited either significantly increased or decreased *irt1* expression. Furthermore, some chlorosis- sensitive lines (A2 and C5) showed greater *irt1* expression levels than other tolerant lines (E1-2 andE7). This is similar to the findings that *irt1* expression in chlorosis-sensitive citrus rootstocks was at least two times higher than the tolerant ones (Martínez-Cuenca et al., 2013). Furthermore, Santos et al. (2015) noted that the expression of *irt1*-like gene was higher in the roots of IFN plants than in the EF ones. The considerably higher gene expression levels (mainly *irt1*) measured in control plant roots and leaves compared to 75% of the plant lines may have been due to a rapid or shock response of control plants to Fe deficiency stress. In contrast, the selected plant lines did not exhibit changes in expression of the genes as they might have adapted to Fe deficiency conditions.

In conclusion, a novel set of EF and IFN potato plant lines has been developed which can be useful in further research, serve as germplasm and/or aid in breeding programs aiming at selecting IDC tolerant potato genotypes. Taken together, the results showed that 37.5% of the putative EF plant lines (A1, B2, and B9) have the capacity to elicit improved morphological attributes including enhanced formation of lateral roots and root hairs under low iron supply conditions. In addition, the EF plant lines were found to exhibit enhanced expression of two Fe homeostasis-related genes, *fer3* in the leaf and *irt1* in the root. These morphological and molecular changes seem to be important for resistance to Fe deficiency stress. The use of these novel plant lines could contribute to enhancement of crop productivity in calcareous soils.

## Author contributions

GB carried out the experiments and wrote the initial drafts of the paper. DL was the senior supervisor and CL was the associate supervisor. DL also wrote and edited the final draft of the paper.

### Conflict of interest statement

The authors declare that the research was conducted in the absence of any commercial or financial relationships that could be construed as a potential conflict of interest.

## Supplementary material

Supplementary Figure 1 Shoot development from selected Fe-efficient potato (cv, “Iwa”) callus lines **(A–E)** and calli grown on control medium **(F)**. Calli were cultured on regeneration media and shoot buds generated after 7 to 14 weeks.

Supplementary Figure 2 Shoot elongation and multiplication of regenerants derived from Fe-efficient potato (cv. “Iwa”) calli selected from 0.1 **(I)**, 0.5 **(II)**, and 5 μM Fe medium **(III)** after 11 weeks (2 subcultures) on half-strength MS medium without PGRs.

Supplementary Figure 3 Stereomicroscopic view of roots of control plant (SK), Fe-efficient (EF) and inefficient (IFN) potato plant lines cultured on Fe-deficient medium. SK had long green roots with few or no lateral roots. EF and IFN plant lines had numerous short roots with lateral roots mostly white to cream in color with some brown regions.

Supplementary Table 1 Scheme for the *in vitro* selection of potato variants for their tolerance to Fe-deficiency.

Supplementary Table 2 Sequences of the oligonucleotide primers used for qPCR.
