# Supplementary figures and images for: Insights into Resistance to Fe Deficiency Stress from a Comparative Study of In Vitro-Selected Novel Fe-Efficient and Fe-Inefficient Potato Plants
Source: Front Plant Sci. 2017 Sep 13;8:1581. doi: 10.3389/fpls.2017.01581 (PMC5601415; doi:10.3389/fpls.2017.01581)

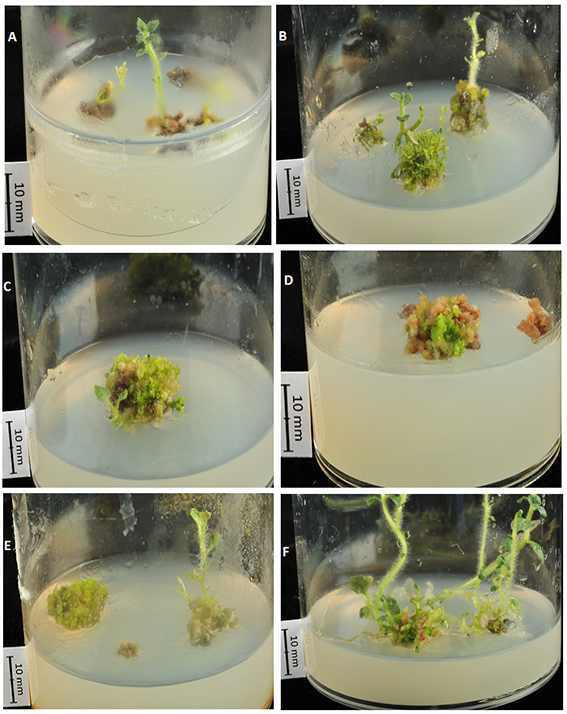

Supplement: Supplementary Figure 1 — Shoot development from selected Fe-efficient potato (cv, “Iwa”) callus lines (A–E) and calli grown on control medium (F). Calli were cultured on regeneration media and shoot buds generated after 7 to 14 weeks. [file Image1.TIF]

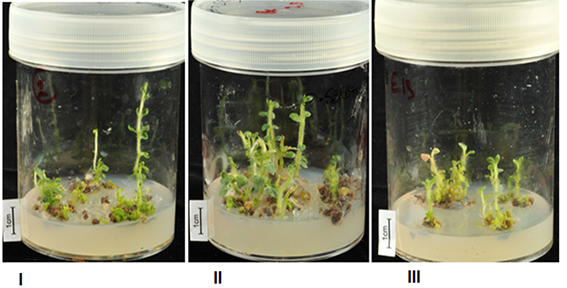

Supplement: Supplementary Figure 2 — Shoot elongation and multiplication of regenerants derived from Fe-efficient potato (cv. “Iwa”) calli selected from 0.1 (I), 0.5 (II), and 5 μM Fe medium (III) after 11 weeks (2 subcultures) on half-strength MS medium without PGRs. [file Image2.TIF]

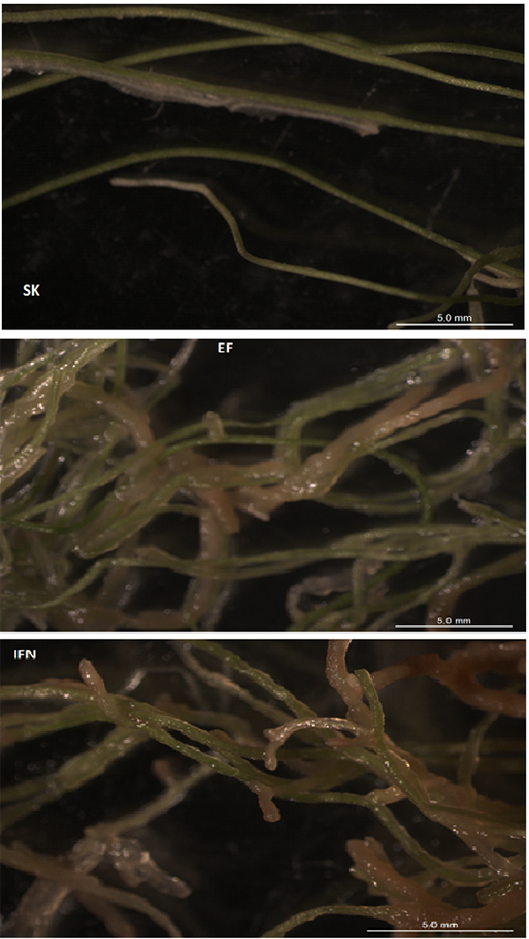

Supplement: Supplementary Figure 3 — Stereomicroscopic view of roots of control plant (SK), Fe-efficient (EF) and inefficient (IFN) potato plant lines cultured on Fe-deficient medium. SK had long green roots with few or no lateral roots. EF and IFN plant lines had numerous short roots with lateral roots mostly white to cream in color with some brown regions. [file Image3.TIF]
