# Supplementary material for: Insights into Resistance to Fe Deficiency Stress from a Comparative Study of In Vitro-Selected Novel Fe-Efficient and Fe-Inefficient Potato Plants
Source: Front Plant Sci. 2017 Sep 13;8:1581. doi: 10.3389/fpls.2017.01581 (PMC5601415; doi:10.3389/fpls.2017.01581)

**Supplementary Table 1.** Scheme for the in vitro selection of potato variants for their tolerance to Fe-deficiency.


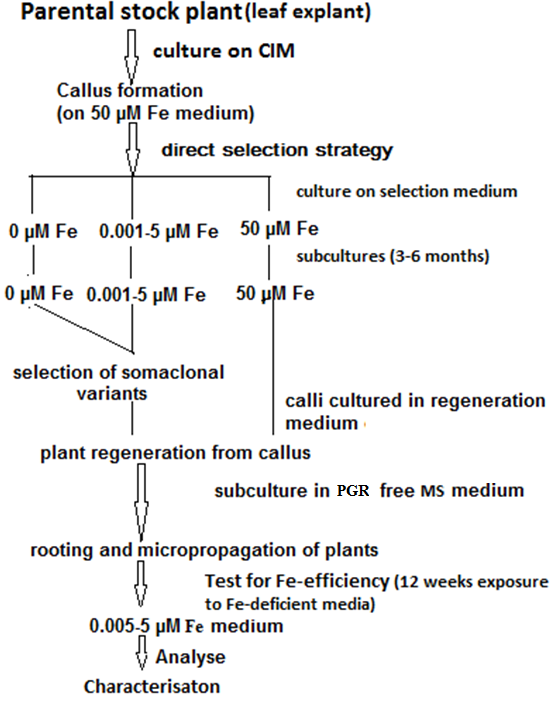


**Supplementary Table*2*.** Sequences of the oligonucleotide primers used for qPCR**.**


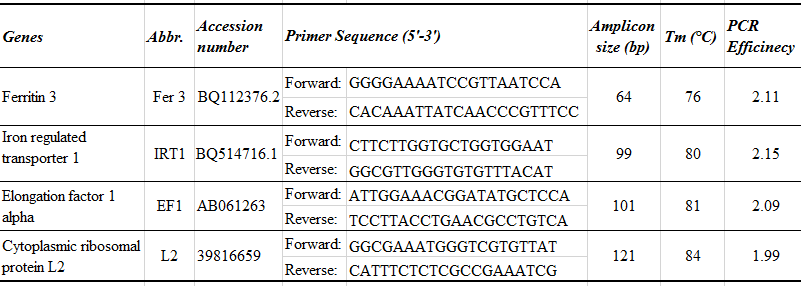

Supplement: Supplementary Table 1 — Scheme for the in vitro selection of potato variants for their tolerance to Fe-deficiency. [file Table1.DOC]
